# Supplementary material for: Psychometric properties of the PHQ-9 depression scale in people with multiple sclerosis: A systematic review
Source: PLoS One. 2019 Feb 19;14(2):e0197943. doi: 10.1371/journal.pone.0197943 (PMC6380554; doi:10.1371/journal.pone.0197943)
Supplement: S2 Fig — (PDF) [file pone.0197943.s002.pdf]

Psychometric properties of the PHQ-9 depression scale in people with multiple sclerosis: a systematic review

Sarah Patrick[1] & Peter Connick[2]
1 – College of Medicine and Veterinary Medicine, University of Edinburgh, Edinburgh, UK.
2 – Centre for Clinical Brain Sciences, University of Edinburgh, Edinburgh, UK.

Background

Multiple sclerosis (MS) affects over 2.5 M people globally. It is a chronic inflammatory and neurodegenerative disease of the central nervous system that commonly presents with sensory, motor, and cognitive impairments. The co-existence of depression in people with MS is increasingly recognised, with a lifetime prevalence >50% and a point prevalence of approximately 25%. Depression is a major determinant of quality of life in people with MS, and is often under-recognised in clinical practice.

A number of tools have been used to quantify the burden of affective symptoms in people with MS, including the Beck Depression Inventory (BDI-II), Hospital Anxiety and Depression Scale (HADS), and the nine-item Patient Health Questionnaire (PHQ-9). The PHQ-9 scale is of particular interest in this context because it is freely available and has been validated across a wide range of clinical populations. The PHQ-9 is a self-report version of the Primary Care Evaluation of Mental Disorders (PRIME-MD), developed in the mid-1990s by Pfizer Inc. It is a self-reported instrument for the evaluation of depressive symptoms over the preceding 2 weeks, and involves 9-items with four responses to indicate the frequency of symptom-experience.

There have been previous reviews of depression scoring tools in people with MS (e.g. a 2016 paper by Hind et al) but there lacks a focused review of the PHQ-9's performance in patients with MS. A more focused systematic review of the PHQ-9's performance in patients with multiple sclerosis is therefore due.

Objective

The performance of the PHQ-9 will be evaluated through the following eight indicators: appropriateness, reliability, validity, responsiveness, precision, interpretability, acceptability, and feasibility (Fitzpatrick et al. Health Technology Assessment. 1998; 2[14]:1-74).

Searches

Evidence will be gathered from the databases 'PubMed', 'Medline' and 'ISI Web of Science'. There will also be hand-searching of references from eligible sources. The full search strategy is recorded on the PROSPERO International prospective register of systematic reviews site (https://www.crd.york.ac.uk/PROSPERO).

Eligibility Criteria
After gathering the evidence, the following eligibility criteria will be applied to the results: The sources must be primary literature, must be in the English language, must be peer-reviewed, and the primary aim of each study must be to evaluate the performance of the PHQ-9 in people with MS (including if the study also evaluates other depression inventories or other conditions). There will be no date restriction on eligibility.

Screening of primary database search: The abstracts of all identified studies will be screened against the eligibility criteria. If no eligibility criteria are breached, the full manuscript will be assessed for eligibility.

Condition or domain being studied
Depression measurement in patients with Multiple Sclerosis (MS)

Participants/ population
The target group in this systematic review are people with MS (any type). Studies with comparison groups (e.g., other conditions – physical or psychological, health subjects) will also be included in the review.

Assessment of Risk of Bias and Data Extraction
A study quality assessment tool will be developed based on STROBE guidelines to evaluate the risk of bias in individual studies.
Initial screening of abstracts will be performed by a single author (SP). Full articles will then be retrieved and eligibility assessment performed in a standardized manner, with a final decision over study inclusion taken in consensus with a second reviewer (PC). A standardised data extraction form will be used, which includes details about the study (authors, year, country), the sample (size, method of recruitment)

Data Synthesis
The findings will be combined narratively.

Dissemination
A paper will be submitted to a scientific journal for publication.

Implications
This study will identify any gaps in the knowledge base around the psychometric performance of the PHQ-9 in MS. This will guide future research prior to wider implementation.

Online access: Details of the protocol for this systematic review were prospectively registered on PROSPERO.

Peter Connick, Sarah Patrick. Psychometric properties of the PHQ-9 depression scale in people with multiple sclerosis: a systematic review. PROSPERO 2017 CRD42017067814

Available from:
http://www.crd.york.ac.uk/PROSPERO/display_record.php?ID=CRD42017067814
