# Supplementary material for: Psychometric properties of the PHQ-9 depression scale in people with multiple sclerosis: A systematic review
Source: PLoS One. 2019 Feb 19;14(2):e0197943. doi: 10.1371/journal.pone.0197943 (PMC6380554; doi:10.1371/journal.pone.0197943)
Supplement: S3 Fig — (PDF) [file pone.0197943.s003.pdf]

Psychometric properties of the PHQ-9 depression scale in people with multiple sclerosis: a systematic review

Supplementary Materials S2: Quality assessment tool for evaluation of manuscripts, based on the STROBE checklist

Instructions: Where all key points are met, 1 point is awarded. Where the study meets most but not all of the applicable criteria, or only part of the relevant information is provided, a score of 0.5 is awarded.

	Criterion		Score [0]/[0.5]/[1]
	INTRODUCTION		
1	**OBJECTIVE: State specific objectives, including any prespecified hypotheses.**<br>• The study should have a clearly stated objective		
	METHODS		
2	**STUDY DESIGN: Present key elements of study design early in the paper.**<br>• The study design should be presented clearly, i.e. retrospective or prospective recruitment, case-control studies, or a sub-study of part of a larger study.<br>• Prospective recruitment to address the study objective is considered preferable and a clear statement of this is needed for 1 point. A retrospective study design will be awarded 0.5.<br>• Where participants are taken from a cohort being used for multiple (sub)studies, a maximum of 0.5 can be awarded. Enough detail should be provided to ensure results are not duplications of other published work.		
3	**SETTING: Describe the setting, locations, and relevant dates, including periods of recruitment, exposure, follow-up, and data collection.**<br>• The dates of recruitment or testing should be provided.<br>• A description of the clinical setting (e.g. tertiary referral centre, multiple district general hospitals etc.) is required<br>• Both the above criteria are necessary for 1 point, either alone will be awarded 0.5.		
4	**PARTICIPANTS: Give the eligibility criteria, and the sources and methods of selection of participants.**<br>• The authors should have clearly stipulated the criteria they used to include (and if applicable, to exclude) subjects into the study. A positive statement of who was sought for recruitment (whether any person with MS, or e.g. only people with a particular clinical phenotype) with relevant exclusion criteria is necessary for 1 mark.<br>• Participants should not be excluded solely on the basis of higher levels of physical disability.		
5	**RECRUITMENT:**<br>• The recruitment should be either a consecutive or random sample of eligible participants. Where this is unclear, the study will be awarded 0.		

	Criterion		Score [0]/[0.5]/[1]
6	**VARIABLES: Clearly define all outcomes, exposures, predictors, potential confounders, and effect modifiers. Give diagnostic criteria, if applicable.**<br>• The tests performed should be specified. Whether results were interpreted relative to a control population or published norms should be clearly stated/described.<br>• Clear definitions as above are required for a score of 1. Where it is unclear, a maximum of 0.5 will be awarded.		
7	• Potential confounding factors, including age, sex, ethnicity, MS Severity, MS Subtype, education and medications should be measured. A score of 1 will be awarded where all these are identified, and 0.5 if ≥4 of them.		
8	**DATA SOURCES/MEASUREMENT: For each variable of interest, give sources of data and details of methods of assessment (measurement). Describe comparability of assessment methods if there is more than one group.**<br>• The person(s) performing the testing should be identified, with their level of training/experience.<br>• Enough data should be provided to replicate the evaluation.<br>• All of the above criteria must be met for a score of 1; where ≥50%, but not all, of the relevant information is presented, the study will be awarded 0.5.		
9	**BIAS: Describe any efforts to address potential sources of bias**<br>• Any depression interviews should be performed blind to the results of the results of the PHQ-9 and this should be clearly stated.<br>• If above is not applicable any attempt to address potential biases must be clearly stated to receive 1 mark.		
10	**STUDY SIZE: Explain how the study size was arrived at.**<br>• A calculation of study size should be provided.		
11	**QUANTITATIVE VARIABLES: Explain how quantitative variables were handled in the analyses. If applicable, describe which groupings were chosen and why.**<br>• Ideally, the full range of scores will be used for the analysis. This should be clearly stated and correlations using the full range of values or correlations by rank will be awarded 1 point<br>• If participants are categorised into groups by results of PHQ-9/other tests, the justification of the group definitions should be provided and boundaries pre-specified. A maximum of 0.5 will be awarded where outcomes are dichotomised (or otherwise grouped) for analysis.		
12	**STATISTICAL METHODS: (a) Describe all statistical methods, including those used to control for confounding. (b) Describe any methods used to examine subgroups and interactions. (c) Explain how missing data were addressed. (d) If applicable, describe analytical methods taking account of sampling strategy. (e) Describe any sensitivity analyses.**<br><br>• Statistical methods should be clearly described.		

	Criterion		Score [0]/[0.5]/[1]
	RESULTS		
13	**PARTICIPANTS: (a) Report numbers of individuals at each stage of study—e.g. numbers potentially eligible, examined for eligibility, confirmed eligible, included in the study, completing follow-up, and analysed. (b) Give reasons for non-participation at each stage. (c) Consider use of a flow diagram.**<br>• Participants recruited but not completing the study should be specified.		
14	**DESCRIPTIVE DATA: (a) Give characteristics of study participants (eg demographic, clinical, social) and information on exposures and potential confounders. (b) Indicate number of participants with missing data for each variable of interest.**<br>• Summary statistics for basic demographic data (age, sex) should be provided. If this is not given, a score of 0 will be awarded.<br>• MS phenotype should be provided – if not given, a maximum of 0.5 can be awarded.<br>• Information on recent steroid use, antidepressant use, and disease-modifying therapy is considered ideal		
15	**OUTCOME DATA: Report numbers of outcome events or summary measures.**<br>• The number of participants with incomplete data for each test should be given. If this is unclear, a maximum of 0.5 can be awarded.		
16	**MAIN RESULTS: (a) Give unadjusted estimates and, if applicable, confounder-adjusted estimates and their precision (eg, 95% confidence interval). Make clear which confounders were adjusted for and why they were included. (b) Report category boundaries when continuous variables were categorized. (c) If relevant, consider translating estimates of relative risk into absolute risk for a meaningful time period.**<br>• Relevant measures should be reported for each of the study objectives.		
